# P31–43, an undigested gliadin peptide, mimics and enhances the innate immune response to viruses and interferes with endocytic trafficking: a role in celiac disease

**DOI:** 10.1038/s41598-018-28830-y

**Published:** 2018-07-17

**Authors:** Merlin Nanayakkara, Giuliana Lania, Mariantonia Maglio, Renata Auricchio, Cristiana De Musis, Valentina Discepolo, Erasmo Miele, Bana Jabri, Riccardo Troncone, Salvatore Auricchio, Maria Vittoria Barone

**Affiliations:** 1 Department of Translational Medical Science (Section of Paediatrics) and ELFID (European Laboratory for the Investigation of Food-Induced Disease), University of Naples, Federico II, Naples, 80131 Italy; 2 Department of Medicine, University of Chicago, Chicago, Illinois 60637 USA

## Abstract

Celiac disease (CD) is an autoimmune disease characterized by inflammation of the intestinal mucosa due to an immune response to wheat gliadins. Some gliadin peptides are resistant to intestinal digestion (e.g., A-gliadin P31–43) and induce a stress/innate immune response, but the reason why they are dangerous in the intestines of patients with CD is unknown. In the present study, P31–43 activated IFN-α, a mediator of the innate immune response in CD, in the intestine of subjects with CD and an enterocyte cell line, CaCo-2. P31–43 cooperated with a viral ligand to activate the TLR7 pathway by interfering with endocytic trafficking. Based on these results, the vesicular pathway regulates the innate/inflammatory response to viral ligands and bioactive dietary peptides. Suggesting that together with viral infections, alimentary proteins able to mimic and potentiate the innate immune response to viruses, can trigger an autoimmune disease such as CD.

## Introduction

Celiac disease (CD) is an autoimmune disease caused by the loss of oral tolerance to gluten, a protein contained in wheat, barley and rye. The disease is characterized by an enteropathy with inflammatory and structural changes that result in remodelling of the small intestinal mucosa. These changes are the consequence of mucosal inflammation resulting from a Th1 response to certain gliadin peptides (e.g., the 33-mer A-gliadin peptide) presented by human leucocyte antigen 2 or 8 (HLA-DQ)^1^ and activation of innate immune pathways. The activation of these pathways may be mediated by several factors, including other gliadin peptides, e.g., A-gliadin peptide P31–43^2^, not presented by HLA-DQ2 or 8^3^. Both the 33-mer and 25-mer (P31–55) containing the peptides P57–68 and P31–43, respectively, are very resistant to hydrolysis by gastric, pancreatic and intestinal proteases. Thus, these peptides are active *in vivo* in the celiac intestine after gluten ingestion^4–6^. Interleukin 15 (IL15) is a major mediator of the proliferative and innate immune response of the celiac intestine to gliadin^7,8^ through cooperation with epidermal growth factor (EGF)^7,9,10^. The mechanisms by which P31–43 might induce the innate immune response and enterocyte proliferation have recently been attributed to effects on the endocytic compartment^7,11^. In both celiac enterocytes and CaCo-2 cells, P31–43 localizes to the early endosomes and delays vesicular trafficking^10–12^. P31–43, but not P57–68, shares sequence similarity with a region of the growth factor regulated tyrosine kinase substrate (HRS) needed for its correct endocytic localization. HRS is a key molecule involved in regulating endocytic maturation that is localized on the membranes of early endocytic vesicles^12^. In CaCo-2 cells, P31–43, but not P57–68, interferes with the correct localization of HRS to early endosomes, delaying the maturation of the endocytic vesicles^12^. Consequently, P31–43 induces two important effects: (a) it delays endocytic maturation and (b) it alters the recycling pathway. A delay in endocytic maturation reduces the degradation of epidermal growth factor receptor (EGFR) and other receptor tyrosine kinases (RTKs), which are endocytosed by these vesicles. This delay prolongs their activation, resulting in increased proliferation, actin remodelling and other biological effects. The alteration of the recycling pathway can direct more IL15 receptor alpha (IL15Rα) to the cell surface, enhancing the trans presentation of IL15/IL15Rα in epithelial cells^7^. Type 1 interferons also play a role in the loss of oral tolerance to gluten in patients with CD. In fact, interferon-alpha (IFN-α) is dysregulated in patients with CD, and IFN-α therapy can induce CD in some genetically susceptible individuals. In addition, rotavirus infections are associated with an increased incidence of CD^13^. Moreover, the combination of viral infections and dietary gliadin causes an enteropathy in normal mice^14^. The cellular rotavirus receptor is Toll-like receptor 7 (TLR7)^15^. TLR7 is an endosomal receptor that specifically recognizes the viral mRNA and is regulated by endosomal trafficking. The signalling pathway initiated by TLR7 when it is engaged by selected viral ligands, including the TLR7-specific ligand loxoribine (LOX), induces the formation of a myeloid differentiation primary response 88 (MyD88)/TLR7 complex that requires endosomal trafficking to be activated. Subsequently, the activated complex induces the phosphorylation of mitogen-activated protein kinase (MAPK) and nuclear factor-κB (NF-κB) activation, ultimately increasing the levels of IFN-α and myxovirus resistance protein 1 (MxA), an antiviral protein that can embed the viral particles^16^. Interestingly, HRS is also a key factor in endosomal TLR7 and Toll-like receptor 9 (TLR9) trafficking; in fact, it is necessary for the ubiquitin-dependent targeting of TLR9 to the lysosomes^17^. Based on these observations, mechanisms regulating vesicular trafficking are central to viral infections response.

In the present study, we investigated whether the A-gliadin peptide P31–43 could mimic and reinforce the IFN-α mediated innate immune response to viruses in biopsies from patients with CD and a gliadin-responsive intestinal cell line, CaCo-2, by interfering with endocytic trafficking.

## Results

### In small intestinal biopsies, MxA and IFN-α are expressed at higher levels in patients with CD on a GCD and the expression of both proteins is induced by P31–43 in patients with CD on a gluten-containing diet (GCD) or a gluten-free diet (GFD)

**Figure 1:**
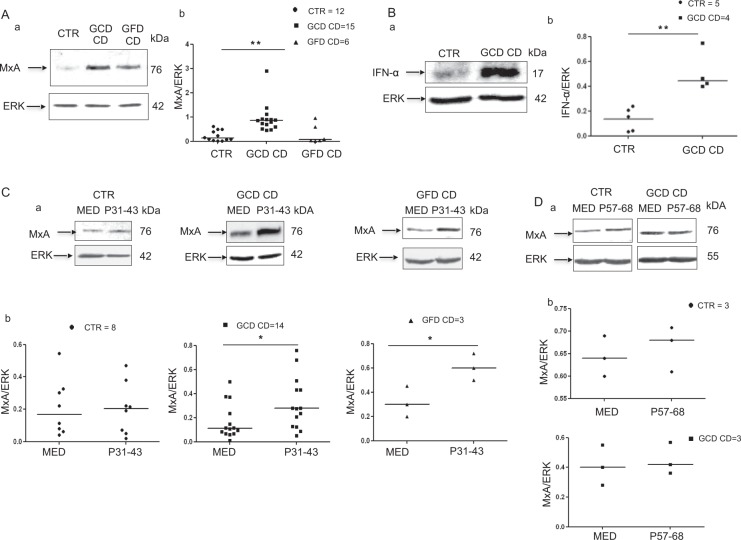
In small intestinal biopsies, MxA and IFN-α were expressed at higher levels in patients with CD on a GCD and were induced by P31-43 in patients with CD on a GCD or GFD. (**A**) MxA was expressed at higher levels in patients with active CD on a gluten-containing diet (GCD CD). (a) Western blot analysis of protein lysates from the intestinal biopsies of patients with CD on a GCD, patients on a gluten-free diet (GFD CD) and controls (CTR). The upper panel was blotted with anti-MxA antibodies and the second panel was blotted anti-ERK antibodies as the loading control. Representative blots are shown. (b) Densitometric analysis. The relative MxA levels were normalized to ERK. The numbers of patients are indicated. Horizontal lines represent the medians. Student’s t-test compared with the control (CTR) samples. **p < 0.01. (**B**) The IFN-α protein was expressed at higher levels in patients with active CD (GCD CD). (a) Western blot analysis of protein lysates from the intestinal biopsies of patients with CD on a GCD and controls, as indicated. The upper panel was blotted with anti-IFN-α antibodies and the second panel was blotted with anti-ERK antibodies as the loading control. Representative blots are shown. (b) Densitometry analysis. The relative amounts of the IFN-α protein were normalized to ERK. The numbers of patients are indicated. Horizontal lines represent the medians. Student’s t-test compared with the control (CTR) samples. **p < 0.01. (**C**) After P31-43 treatment, MxA expression was increased in the biopsies from subjects with CD on a GCD or GFD. (a) Western blot analysis of protein lysates from the intestinal biopsies of patients with CD on a GCD or GFD and the controls that had been cultivated for 24 h with and without P31-43. The upper panel was blotted with anti-MxA antibodies and the second panel was blotted with anti-ERK antibodies as the loading controls. Representative blots are shown. (b) Densitometric analysis. The relative MxA levels were normalized to ERK. The numbers of patients are indicated. Horizontal lines represent the medians. Student’s *t*-test compared with the untreated (MED) sample, *p < 0.05. (**D**) After P57-68 treatment, MxA expression was not increased in the biopsies of subjects with CD on a GCD and CTR. (a) Western blot analysis of protein lysates from the intestinal biopsies of patients with CD on a GCD and controls that had been cultivated for 24 h with and without P57-68. The upper panel was blotted with anti-MxA antibodies and the second panel was blotted with anti-ERK antibodies. Representative blots are shown. (b) Densitometric analysis. The relative MxA levels were normalized to ERK. The numbers of patients are indicated. Horizontal lines represent the medians. Student’s *t*-test compared with the untreated (MED) sample.

The IFN-α pathway was activated in CD biopsies in previous reports^13,18^. We confirmed these observations by analysing levels of the MxA protein in biopsies from patients with CD on a GCD or GFD. MxA levels were only increased in patients with CD on a GCD (Fig. 1A). Specifically, the ratio of MxA to glyceraldehyde 3-phosphate dehydrogenase (GAPDH) proteins increased from 0.2 ± 0.2 in the controls to 0.9 ± 0.59 (*p* = 0.006) in patients on a GCD, whereas it was only 0.28 ± 0.39 in the biopsies from patients on a GFD. In addition, levels of the IFN-α protein were increased in patients with CD on a GCD (Fig. 1B). *In vitro* P31–43, but not P57–68, treatment increased MxA levels in cultures of the intestinal biopsies from patients with CD on either diet. Values for cultures obtained from subjects on a GCD (0.3 ± 0.14, *p* = 0.038) and GFD (0.6 ± 0.10, *p* = 0.039) were greater than values for cultures of the untreated biopsies (0.16 +/− 0.14 and 0.3 +/− 0.12, respectively). Levels of the IFN-α 7 and 17 mRNAs were also increased after treatment with P31–43 in biopsies from patients with CD on a GCD (data not shown). In cultured biopsies from the control subjects (CTR), P31–43 had no effect on the levels of the MxA protein (Fig. 1C) or IFN-α 7 and 17 mRNAs (data not shown). Thus, the IFN-α pathway was activated by the gliadin peptide P31–43 in the small intestine of patients with CD.

### Similar to the viral ligand LOX, P31–43 activates the TLR7 pathway

**Figure 2:**
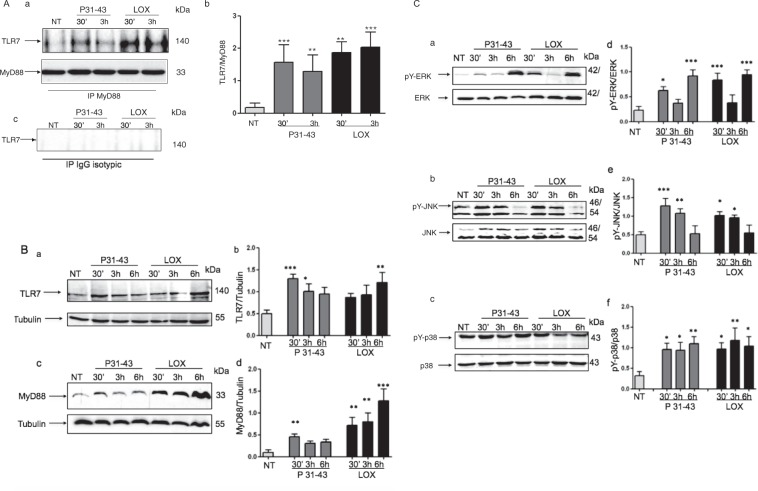
Activation of the TLR7 pathway by the viral ligand LOX and gliadin peptide P31–43 in CaCo-2 cells. (**A**) Similar to the viral ligand LOX, P31–43 increased the formation of the MyD88/TLR7 complex. (a) Western blot analysis of immunoprecipitated MyD88 in CaCo-2 cells. The upper panel was blotted with anti-TLR7 antibodies and the second panel was blotted with anti-MyD88 antibodies. The treatments and times are indicated. The results are representative of 3 independent experiments. (b) Densitometry analysis. The relative amount of TLR7 in the complex was normalized to immunoprecipitated MyD88. Columns represent the means and bars represent the standard deviations of 3 independent experiments. NT = not treated. One-way ANOVA, Bonferroni-corrected. **p < 0.01, ***p < 0.001. (c) Immunoprecipitation control. Western blot analysis of immunoprecipitated isotype-control IgG in untreated CaCo-2 cells and cells treated with P31–43 or LOX and blotted for anti-TLR7. No band was immunoprecipitated by the isotype-control IgG. The treatments and times are indicated. The results are representative of 3 independent experiments. (**B**) Levels of the TLR7 and MyD88 proteins increased in cells treated with the viral ligand LOX or P31–43. Western blot analysis of protein lysates from CaCo-2 cells treated with the indicated compounds. The upper panels were blotted with anti-TLR7 (a) and anti-MyD88 antibodies (c) the second panels in both a and c were blotted with anti-tubulin antibodies as a loading control. The results are representative of 3 independent experiments. (b and d) Densitometric analyses. The relative amounts of TLR7 (b) and MyD88 (d) were normalized to the tubulin levels. The treatments and times are indicated. Columns represent the means and bars represent the standard deviations of 3 independent experiments. NT = not treated. One-way ANOVA, Bonferroni-corrected. *p < 0.05, **p < 0.01, ***p < 0.001. (**C**) Similar to the viral ligand LOX, P31–43 activated MAPKs. (a–c). Western blot analysis of protein lysates from CaCo-2 cells treated with the indicated compounds. The upper panels were blotted with antibodies against the phosphorylated forms of ERK (a) pY-ERK), JNK (b) pY-JNK) and p38 (c) pY-p38), whereas the second panels were blotted for the non-phosphorylated forms of ERK, JNK and p38, respectively. The results are representative of 3 independent experiments. (d–f) Densitometric analyses. The relative amounts of pY-ERK, pY-JNK and pY-p38 were normalized to levels of ERK (d) JNK (e) and p38 (f) respectively. The treatments and times are indicated. Columns represent the means and bars represent the standard deviations of 3 independent experiments. NT = not treated. One-way ANOVA, Bonferroni-corrected. *p < 0.05, **p < 0.01, ***p < 0.001.

We investigated the effects of the gliadin peptide P31–43 on CaCo-2 cells, an intestinal epithelial cell line that is responsive to gliadin, to obtain a better understanding of the mechanisms by which this peptide activates the IFN-α pathway. We first investigated the specificity and toxicity of the P31–43 peptide in CaCo-2 cells. The specificity of the effects of P31–43 was confirmed by examining extracellular signal-regulated kinase (ERK) activation in CaCo-2 cells after treatment with permuted P31–43 (alanine scanning). Most of the mutations reduced ERK phosphorylation (pY-ERK) (Supplemental Fig. 1A). The toxicity of P31–43 was analysed using Trypan blue staining, a vital colorant. As shown in Supplemental Fig. 1B, treatment with 100 μg/ml P31–43 and P57–68 peptides had no effect on cell viability. Interestingly, the amount of P31–43 used in our experiments was not substantially different from the amount that is presumably generated in the intestine during the digestion of wheat flour^6^. Moreover, a dose response assay using pY-ERK levels as a readout of the effects of P31–43 and P57–68 showed that treatment with 50 and 100 μg/ml P31–43 activated ERK. P57–68 did not activate ERK at any concentration tested (Supplemental Fig. 1C). We compared the effects of P31–43 and the viral ligand LOX on the activation of the TLR7 pathway in CaCo-2 cells. LOX specifically activates the MyD88-dependent TLR7 signalling pathway via TLR7^16^. Moreover, CaCo-2 cells in general and, specifically, our clone do not express the other endosomal receptor, TLR9 (^19^ and not shown), confirming that the CaCo-2 cell line is a good model with which to study TLR7 activation in epithelial intestinal cells. We compared the effects of P31–43 on the activation of the TLR7 pathway in CaCo-2 cells with those of LOX, beginning with the formation of the MyD88/TLR7 complex and subsequent activation of mitogen-activated protein kinases (MAPKs). Figure 2A shows the increase in formation of the MyD88/TLR7 complex induced by the viral ligand LOX and by P31–43 after stimulation for 30 min or 3 h. The amount of TLR7 in the complex with MyD88 in CaCo-2 cells increased from 0.16 ± 0.13 in the untreated sample to 1.8 ± 0.33 and 2 ± 0.47 in samples treated with LOX for 30 min or 3 h, respectively. Similarly, the gliadin peptide P31–43 induced a comparable increase in the level of TLR7 in the complex with MyD88 at 30 min (1.5 ± 0.53) and 3 h (1.2 ± 0.49). We next investigated the effects of LOX and P31–43 on the levels of the TLR7 and MyD88 proteins and found that TLR7 levels were significantly increased in cells treated with LOX for 6 h, (*p* < 0.01) or P31–43 for 30 min (*p* < 0.001) or 3 h (*p* < 0.05). MyD88 levels were also increased at all time points after LOX stimulation. In contrast, after P31–43 treatment, increases in the MyD88 levels were statistically significant only after 30 min of stimulation (Fig. 2B). The time-dependent differences in the effects of LOX and P31–43 potentially suggest that the mechanisms through which each stimulus exerts its effects are not the same. The MyD88/TLR7 complexes induced both by LOX and P31–43 could activate downstream signalling by activating MAPKs, ERK, c-Jun N-terminal kinase (JNK) and protein 38 (p38). Specifically, as shown in Fig. 2C, LOX and P31–43 induced similar increases in the levels of the phosphorylated forms of pY-ERK, JNK (pY-JNK) and p38 (pY-p38) with very similar kinetics. Based on these data, P31–43 activates the same TLR7 pathway in intestinal epithelial cells that is specifically activated by the viral ligand LOX.

### P31–43 and the viral ligand LOX induce the expression of markers of the activation of inflammation and innate immunity

**Figure 3:**
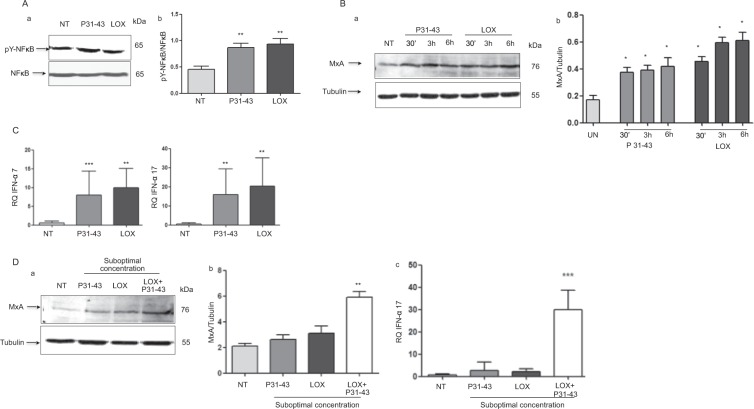
P31–43 and LOX induce the expression of markers of inflammation and innate immunity in CaCo-2 cells. P31–43 and the viral ligand LOX activated the NF-κB and IFN-α pathways. (**A**) Western blot analysis of protein lysates from CaCo-2 cells treated with the indicated compounds for 30′. (a) The upper panel presents the blot of the phosphorylated form of NF-κB (pY-NF-κB) and the second panel presents the blot of the total NF-κB protein. The results are representative of 3 independent experiments. (b) Densitometric analysis. The relative pY-NF-κB levels were normalized to the total NF-κB levels. The treatments are indicated. Columns represent the means and bars represent the standard deviations of 3 independent experiments. NT = not treated. Student’s t-test compared with the NT sample. **p < 0.01. (**B**) (a) Western blot analysis of protein lysates from CaCo-2 cells treated with the indicated compounds. The upper panel was blotted with anti-MxA antibodies and the second panel was blotted with anti-tubulin antibodies as the loading control. The results are representative of 3 independent experiments. (b) Densitometric analysis. The relative MxA levels were normalized to tubulin. The times and treatments are indicated. Columns represent the means and bars represent the standard deviations of 3 independent experiments. NT = untreated. One-way ANOVA, Bonferroni-corrected. *p < 0.05. (**C**) Both P31–43 and LOX increased levels of the IFN-α 7 and 17 mRNA. Quantitative PCR analysis of the IFN-α 7 and 17 mRNAs in CaCo-2 cells treated with P31–43 and LOX overnight (ON). RQ = relative quantities of the IFN-α 7 and 17 mRNAs. Columns represent the means and bars represent the standard deviations of 3 independent experiments. NT = untreated. Student’s t-test compared with the NT sample. **p < 0.01, ***p < 0.001. (**D**) The combined application of suboptimal concentrations of P31–43 and LOX induced MxA and IFN-α expression. (a) Western blot analysis of protein lysates from CaCo-2 cells treated with suboptimal concentrations of P31–43 (20 μg/ml) and LOX (250 μM). The upper panel was blotted with anti-MxA antibodies and the second panel was blotted with anti-tubulin antibodies as the loading control. The results are representative of 3 independent experiments. (b) Densitometric analysis. The relative MxA levels were normalized to tubulin. The treatments are indicated. Columns represent the means and bars represent the standard deviations of 3 independent experiments. Student’s t-test compared to the untreated (NT) sample. **p < 0.01. (c) Quantitative PCR analysis of the IFN-α 17 mRNA in CaCo-2 cells after an ON treatment with suboptimal concentrations of P31–43 and LOX. RQ = relative quantity. Columns represent the means and bars represent the standard deviations of 3 independent experiments. Student’s *t*-test compared with the untreated (NT) sample. ***p < 0.001.

Activation of TLR7 and 9 leads to an innate immune response (increased IFN-α levels) and an inflammatory response (NF-κB activation)^20^. LOX and P31–43 treatments increased NF-κB phosphorylation in CaCo-2 cells from 0.45 ± 0.06 in control cells to 0.93 ± 0.1 and 0.86 ± 0.08, respectively, as shown in Fig. 3A. We investigated the levels of the MxA protein, a downstream effector of IFN-α activation to follow the activation of the IFN-α pathway. Both LOX and P31–43 treatments induced a statistically significant increase in levels of the MxA protein at all time points investigated (Fig. 3B). The control gliadin peptide P57–68, which does not delay vesicular trafficking^12^, was not effective at promoting the formation of the TLR7/MyD-88 complex, increasing the levels of the TLR7, MyD-88, MxA and IFN-α proteins or activating NF-κB (Supplemental Fig. 2). The levels of the IFN-α 7 and 17 mRNAs were also analysed after P31–43 and LOX treatments. Levels of the IFN-α 7 and 17 mRNAs were increased to similar extents after an overnight (ON) treatment with LOX or P31–34 (Fig. 3C). Cooperation between viral ligands and gliadin peptides induces enteropathy in mice^14^. We investigated whether LOX and P31–43 cooperate to induce the activation of the IFN-α pathway in our system. As shown in Fig. 3D, stimulation of CaCo-2 cells with suboptimal concentrations of LOX or P31–43 alone did not increase levels of either the MxA protein or IFN-α mRNA. However, when LOX and P31–43 were added together at suboptimal concentrations, levels of both the MxA protein (*p* < 0.01) and IFN-α mRNA (*p* < 0.001) were increased, indicating the synergistic cooperation between the viral ligand and gliadin peptide.

### The increase in MxA levels observed in response to P31–43 and LOX treatments depends on the formation of the MyD88/TLR7 complex

**Figure 4:**
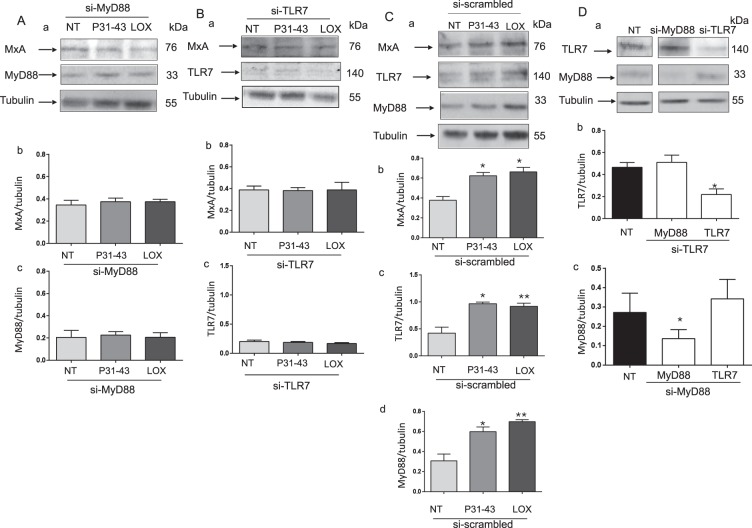
The increase in MxA levels induced by the P31–43 and LOX treatments depends on the formation of the MyD88/TLR7 complex in CaCo-2 cells. (**A**) Silencing of MyD88 (si-MyD88) prevented the increase of the MxA and MyD88 protein levels induced by 30′ of P31–43 or LOX treatment. (a) Western blot analysis of levels of the MxA and MyD88 proteins in cells transfected with si-MyD88. The upper panel was blotted with anti-MxA antibodies, the second panel was blotted with anti-MyD88 antibodies and the third panel was blotted with anti-tubulin antibodies. The treatments are indicated. The results are representative of 3 independent experiments. (b and c) Densitometric analyses. Columns represent the means and bars represent the standard deviations of 3 independent experiments. NT = not treated. Student’s *t*-test compared with the untreated (NT) sample. (**B**) Silencing of TLR7 (si-TLR7) prevented the increase in the levels of the MxA and TLR7 proteins induced by 30′ of P31–43 or LOX treatment. (a) Western blot analysis of levels of the MxA and TLR7 proteins in cells transfected with si-TLR7. The upper panel was blotted with anti-MxA antibodies, the second panel was blotted with anti-TLR7 antibodies and the third panel was blotted with anti-tubulin antibodies. The treatments are indicated. The results are representative of 3 independent experiments. b and c) Densitometric analyses. Columns represent the means and bars represent the standard deviations of 3 independent experiments. NT = not treated. Student’s *t*-test compared with the untreated (NT) sample. (**C**) A control scrambled siRNA (si-scrambled) did not prevent the increase in the levels of the MxA, TLR7 and MyD88 proteins after 30′ treatments with P31–43 or LOX. (a) Western blot analysis of levels of the MxA, TLR7 and MyD88 proteins in cells transfected with si-scrambled. The upper panel was blotted with anti-MxA antibodies, the second panel was blotted with anti-TLR7 antibodies, the third panel was blotted with anti-MyD88 antibodies, and the fourth panel was blotted with anti-tubulin antibodies. The treatments are indicated. The results are representative of 3 independent experiments. (b,c and d) Densitometric analyses. Columns represent the means and bars represent the standard deviations of 3 independent experiments. NT = untreated. Student’s *t*-test compared with the untreated (NT) sample. *p < 0.05, **p < 0.01. (**D**) The MyD88 and TLR7 siRNAs were specific for their own targets. (a) Western blot analysis of levels of the MyD88 or TLR7 proteins before and after silencing with TLR7 or MyD88 siRNAs. The upper and middle panels were blotted with anti-TLR7 and anti-MyD88 antibodies, respectively, and the lower panel was blotted with anti-tubulin antibodies. The results are representative of 3 independent experiments. (b and c) Densitometric analyses. The relative MyD88 and TLR7 levels were normalized to tubulin. Columns represent the means and bars represent the standard deviations of 3 independent experiments. NT = not treated. Student’s *t*-test compared with the untreated (NT) sample. *p < 0.05.

The engagement of TLR7 by the viral ligand LOX induces downstream signalling pathways that depend on the formation of the MyD88/TLR7 complex^16^. We have confirmed this model in the present study, by showing that the increase in the level of the MxA protein observed after LOX treatment was prevented by silencing of either the MyD88 or TLR7 protein. The P31–43-induced increase in the level of the MxA protein was also prevented by MyD88 or TLR7 silencing, as shown in Fig. 4A and B, indicating that the activation of the IFN-α pathway depended on the TLR7 activation in response to both LOX and P31–43 treatments. A control scrambled siRNA had no effect on the P31–43- or LOX-induced increases in levels of the MxA, TLR7 and MyD88 proteins (Fig. 4C). Silencing of TLR7 and MyD88 proteins reduced the levels of the TLR7 and MyD88 proteins. Silencing of TLR7 had no effect on levels of the MyD88 protein, and silencing of MyD88 had no effect on levels of the TLR7 protein (Fig. 4D).

As shown in our previous studies, P31–43 activates EGFR and other receptors by delaying their intracellular decay through its effect on vesicular trafficking^9,10,12^. Interestingly, the EGFR receptor complexed with TLR7 after treatment with LOX or P31–43, as shown in Supplemental Fig. 3. This finding confirms that EGFR transduces signals from several different receptors.

### P31–43 and LOX treatments delay TLR7 trafficking in the early endocytic compartment

Viruses can interfere with vesicular trafficking at various levels during the infection of a cell^20^. In particular, the MyD88/TLR7 complex that forms in response to the viral ligand LOX requires endosomal trafficking for activation^16^. Moreover, P31–43 is known to delay endocytic trafficking from early to late endocytic vesicles^10–12^. We compared the trafficking of TLR7 from early vesicles (early endocytic antigen, EEA1-positive) to late vesicles (lysosome-associated membrane proteins, LAMP 2-positive) after LOX and P31–43 stimulation. TLR7 exhibited greater co-localization with EEA1 and less co-localization with LAMP2 in samples treated with either LOX and P31–43 than in the untreated sample (Supplemental Fig. 4). We evaluated the trafficking of TLR7 after P31–43 treatment in time-course experiments. P31–43, but not P57–68, delayed TLR7 trafficking in EEA1-positive vesicles after 3 h of treatment (Supplemental Fig. 5). In conclusion, both the viral ligand and the gliadin peptide P31–43 delayed TLR7 trafficking to the early endocytic compartment.

### The delay of the maturation from early to late vesicles activated the TLR7 pathway

P31–43 delayed endocytic maturation by interfering with the correct localization of HRS on endocytic vesicles^10–12^. HRS is a key molecule required for the maturation of early to late endocytic vesicles^21^. We tested the hypothesis that the delay in the maturation of early to late vesicles could, *per se*, activate the TLR7 pathway. We tested this hypothesis by inducing a delay in the maturation of the vesicles through the silencing of the HRS protein (si-HRS)^12^ and analysed the trafficking of TLR7 to the early and late compartments. Under these conditions, more TLR7 was co-localized with the EEA1**-**positive early vesicles (Supplementary Fig. 4) and less with the LAMP2-positive late vesicles, similar to the effects described after LOX and P31–43 treatments (Supplementary Fig. 4).

**Figure 5:**
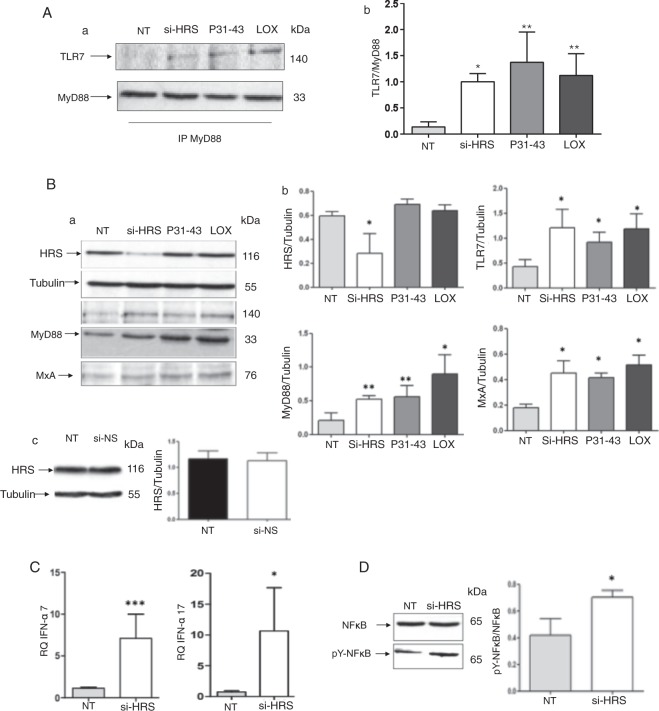
The HRS silencing-mediated delay of the maturation of endocytosis activates the TLR7 pathway in CaCo-2 cells. (**A**) Silencing of HRS and 30′ treatments with P31–43 or the viral ligand LOX increase the formation of the MyD88/TLR7 complex. (a) Western blot analysis of immunoprecipitated MyD88 in CaCo-2 cells. The upper panel was blotted with anti-TLR7 antibodies and the second panel was blotted with anti-MyD88 antibodies. The treatments are indicated. The results are representative of 3 independent experiments. (b) Densitometric analysis. The relative amounts of TLR7 in the complex were normalized to the levels of immunoprecipitated MyD88. The treatments are indicated. Columns represent the means and bars represent the standard deviations of 3 independent experiments. NT = not treated. Student’s t-test, compared with the untreated NT sample. *p < 0.05; **p < 0.01. (**B**) Silencing of HRS or treatment with P31–43 or the viral ligand LOX for 30′ increased the expression of TLR7, MyD88 and MxA. (a) Western blot analysis of protein lysates from CaCo-2 cells that had been treated with the indicated compounds. The upper panel was blotted with anti-HRS antibodies and the second panel was blotted with anti-tubulin antibodies as the loading control. The third, fourth and fifth panels were blotted with antibodies against TLR7, MyD88 and MxA, respectively. The results are representative of 3 independent experiments. (b) Densitometric analysis. The relative HRS, TLR7, MyD88 and MxA levels were normalized to tubulin. The treatments are indicated. Columns represent the means and bars represent the standard deviations of 3 independent experiments. NT = not tretaed. Student’s t-test, compared with the NT sample. *p < 0.05, **p < 0.01. (c) A control non-specific siRNA was ineffective at inhibiting HRS expression. Western blot analysis of levels of the HRS protein before and after silencing with a non-specific siRNA (si-NS). The upper panel was blotted with anti-HRS antibodies and the second panel was blotted with anti-tubulin antibodies. The results are representative of 3 independent experiments. Densitometric analysis. The relative HRS levels were normalized to tubulin. Columns represent the means, and bars represent the standard deviations of 3 independent experiments. NT = not treated. Student’s *t*-test. (**C**) Increased levels of the IFN-α 7 and 17 mRNAs were detected in CaCo-2 cells transfected with si-HRS. RQ = relative quantities of IFN-α 7 and 17 mRNAs. Columns represent the means and bars represent the standard deviations of 3 independent experiments. NT = not treated. Student’s t-test compared with the NT sample, *p < 0.05, ***p < 0.001. (**D**) HRS silencing increased the levels of pY-NF-κB. (a) Western blot analysis of protein lysates from CaCo-2 cells that had been treated with the indicated compounds. The lower panel shows the levels of the phosphorylated form of NF-κB (pY-NF-κB), and the upper panel shows the levels of the total NF-κB protein. The results are representative of 3 independent experiments. (b) Densitometryic analysis. The relative pY-NF-κB levels were normalized to the total NF-κB levels. Columns represent the means and bars represent the standard deviations of 3 independent experiments. NT = not treated. Student’s t-test compared with the NT sample, *p < 0.05.

We next studied the effects of si-HRS on the activation of the TLR7 pathway. As read outs of TLR7 activation, we analysed the formation of the MyD88/TLR7 complex (Fig. 5A), expression of MxA (Fig. 5B) and IFN-α (Fig. 5C) and the activation of NF-κB (Fig. 5D) after HRS silencing. Similar to the effects of the gliadin peptide P31–43 and LOX, the amount of TLR7 in complexes with MyD88 was significantly increased compared to the untreated sample (*p* = 0.03). Levels of the MxA (*p* = 0.02), IFN-α 7 (*p* = 0.0009) and 17 (*p* = 0.04) mRNAs and the phosphorylated form of NF-κB (p = 0.03) were increased after HRS silencing (Fig. 5). Additionally, levels of both the TLR7 and MyD88 proteins were increased after si-HRS treatment (Fig. 5B). Control si-mRNA had no effect on HRS expression (Fig. 5Bc). In conclusion, the alterations in vesicular trafficking induced by si-HRS activated the TLR7 pathway and the IFN-α-mediated immune response, as well as NF-κB-mediated inflammation.

## Discussion

In the present study, the undigested A-gliadin peptide P31–43 activated the IFN-α pathway in the intestines of patients with CD and in the gliadin-responsive^7,9,10,12,22–25^ enterocyte cell line, CaCo-2.

P31–43 could induced the IFN-α-mediated innate immune response in CaCo-2 cells by activating the TLR7 pathway, similar to the viral ligand LOX. Both the viral ligand LOX and P31–43 delayed the trafficking of TLR7 from the early endocytic compartment. Moreover, P31–43 and LOX induced increased complex formation and levels of the TLR7 and MyD88 proteins, subsequently activating MAPKs, the innate immune response (increase in levels of IFN-α and the antiviral protein MxA), and an inflammatory response (increased phosphorylation of NF-κB). Finally, MyD88 and TLR7 silencing prevented complex formation and downstream pathway activation after either LOX or P31–43 treatment, indicating that complex formation is necessary for both stimuli to exert their effects. Interestingly, LOX and P31–43 cooperated to induce the activation of the IFN-α pathway. Specifically, when LOX and P31–43 were applied together at suboptimal concentrations, levels of both the MxA protein and IFN-α mRNA were increased. Cooperation between viral ligands and gliadin peptides, including P31–43, has been shown to induce enteropathy in mice^14,26^.

Subsequently, we tested the hypothesis that P31–43 activated the IFN-α pathway by interfering with endocytic trafficking. This hypothesis is based on two main points. First, P31–43 delayed the maturation of the early to late endocytic vesicles by interfering with the correct endocytic localization of HRS^12^. Second, TLR7 is an endosomal receptor that responds to viral ligands by activating the IFN-α pathway, and its activation is regulated by vesicular trafficking^16,17^.

These findings imply that vesicular trafficking plays a central role in modulating the innate immune activation in the host-pathogen response.

We induced a delay in the maturation of endocytic trafficking by silencing HRS, a master regulator of endocytic maturation^12^, to confirm the role of the delay in vesicular trafficking in IFN-α activation. HRS silencing mimicked all the measured effects of P31–43 on the TLR7 pathway: the delay in TLR7 trafficking to the early vesicles, the increases in the formation of the MyD88/TLR7 complex and protein levels, and the increases in levels of the IFN-α 7/17 mRNAs and MxA protein and NF-κB activation. These data are consistent with the hypothesis that the mechanism by which P31–43 activates the TLR7 pathway is mediated by a delay in endocytic trafficking. In fact, endocytosis exerts many effects on signalling. The endocytic pathway and signalling pathways are regulated reciprocally^27^. The TLR7/MyD88 complex could form in the delayed endocytic vesicles either because the delay in maturation alters the lipid composition and/or pH of vesicles or by other mechanisms present in cells^28^.

Type 1 interferons play a role in the loss of oral tolerance to gluten in patients with CD^13,18^. In the present study, we confirmed that IFN-α is a mediator of the innate immune response in patients with CD by showing an increase in the levels of the IFN-α and MxA proteins and the IFN-α 7 and 17 mRNAs in the intestinal biopsies from patients with CD on a GCD. The activation of the IFN-α pathway may have been induced by dietary gluten. In fact, the gliadin peptide P31–43 activated the IFN-α pathway in biopsies from patients with CD on a GFD and GCD, but not biopsies from controls, indicating a specific effect of gliadin on the intestines of patients with CD.

Interestingly, we observed a constitutive alteration of the vesicular trafficking in cells from patients with CD that is characterized by a delay in the maturation of early to the late vesicles, which predisposed the celiac cells to the activation of the innate immune response to gliadin (manuscript submitted). This finding could explain the specific effect of P31–43 on the activation of the IFN-α pathway in biopsies from patients with CD.

Based on the results presented here, viral infections and alimentary proteins, which are able to mimic and potentiate the innate immune response to viruses, trigger an autoimmune disease. In addition to CD, other autoimmune diseases result from the interactions of several factors, including genetics and the environment. Type 1 interferon activation is linked to autoimmunity not only in CD but also in several other human diseases, including type 1 diabetes^29^. Unlike CD, genetic polymorphisms that directly activate the type 1 interferon pathways are present in some of these diseases, although these alterations alone are not sufficient to induce the disease. The penetrance of these genetic profiles is determined by mostly unknown environmental factors, including disturbances in the microbiota and/or frequent viral infections^29^. In the present study, another environmental factor may be an alimentary protein, such as gliadin. Thus, gluten itself might be a risk factor for these conditions^30^.

## Methods

### Cell culture, materials and treatments

CaCo-2 cells were grown for 5–6 days in Dulbecco’s Modified Eagle’s Medium (DMEM) (GIBCO, San Giuliano Milanese, Italy) supplemented with 10% fetal calf serum (FCS, GIBCO), 100 units/ml of penicillin-streptomycin (GIBCO), and 1 mM glutamine (GIBCO). The medium was changed every two days. Lipopolysaccharide (LPS)-free synthetic peptides (Inbios, Naples, Italy) (>95% pure, evaluated by matrix-assisted laser desorption/ionization time-of-flight mass spectrometry) were obtained using Ultrasart-D20 filtration (Sartorius AG, Gottingen, Germany). The level of LPS in these peptides was below the detection threshold (i.e., <0.20 EU/mg), as assessed using the QCL-1000 kit (Cambrex Corporation, NJ). The sequence of P31–43 was LGQQQPFPPQQPY and that of P57–68 was QLQPFPQPQLPY. The peptides were used at a concentration of 100 μg/ml^10^. The guanosine analog/TLR7 ligand loxoribine (7-allyl-7,8 dihydro-8-oxo-guanosine) from InvivoGen (San Diego, CA, USA) was used at a concentration of 1 mM^16^.

### EEA1 and Lamp staining

CaCo-2 cells were seeded on glass coverslips for 2 days. Than they were stained for 1 hour at room temperature with anti-TLR7 and anti EEA1 or LAMP2 antibody after fixation with 3% paraformaldeide for 5 minutes at room temperature and permeabilization with Triton (Biorad, Milan, Italy) 0.2% for 3 minutes at room temperature. Secondary antibodies Alexa-488 conjugated (Invitrogen, Milan, Italy) anti-rabbit for TLR; anti-goat Alexa-546 (Invitrogen, Milan, Italy) for EEA1; anti-mouse Alexa-546 (Invitrogen, Milan, Italy) for LAMP2 were added to the coverslips for 1 hour at room temperature. Nuclei were stained with Topro-3 iodide conjugate (642/661) (Invitrogen, Milan, Italy). The coverslips were then mounted on glass slides and observed by confocal microscope (LSM 510 Zeiss). Twenty to 30 cells were observed in each sample. Images were generated with a confocal microscope LSM 510 Zeiss. Co-localization analysis was performed with AIS Zeiss software. Magnification of the micrographs was the same for all the figures shown (63x objective, 2X zoom).

### Colocalization analysis

Samples were examined with a Zeiss LSM 510 laser scanning confocal microscope. We used Argon/2 (458, 477, 488, 514 nanometers) and HeNe1 (543 nanometers) and HeNe2 (633 nanometers) excitation lasers, which were switched on separately to reduce cross-talk of the three fluorochromes. The green and the red emissions were separated by a dichroic splitter (FT 560) and filtered (515-to 540-nm band-pass filter for green and >610-nm long pass filter for red emission). A threshold was applied to the images to exclude about 99% of the signal found in control images. The weighted co-localization coefficient represents the sum of intensity of co-localizing pixels in channels 1 and 2 as compared to the overall sum of pixel intensities above threshold. This value could be 0 (no co-localization) or 1 (all pixels co-localize). Bright pixels contribute more than faint pixels. The co-localization coefficient represents the weighted colocalization coefficients of Ch1 (red) with respect to Ch2 (green) for each experiment^25,26^.

### Silencing experiments

Silencing experiments were performed with two different silencing mRNAs for HRS (Hs HRS 5 and Hs HRS 6), MyD88 (Hs MYD88 2 and Hs MYD88 8), TLR7 (Hs TLR7 6 and Hs TLR7 8) with similar results. Non-specific siRNA (MAPK1) was used for transfection efficiency (not shown) and scrambled mRNA sequences was used to test specificity of the silencing (All Stars Negative). All silencing mRNAs were all purchased from QIAGEN, Milan, Italy. Transfections were carried out using the HIPerFect Transfection Reagent following the manufacturer’s instructions (QIAGEN, Milan, Italy).

Briefly, CaCo-2 cells were incubated in standard growth conditions, and 6 μg of siRNA was diluted in 1 ml of culture medium without serum to give a final siRNA concentration of 50 nM. Twenty microliters of HIPerFect Transfection reagent were added to the siRNA mixture with vortexing, and the transfection mixture was added drop wise onto the cells and **i**ncubated for 72 h. The cells were then processed for immunoprecipitation and for Western blot (WB) analysis.

### Western blotting

CaCo-2 cells grown as before were stimulated with P31–43 or LOX for various times at 37 °C. The cells were washed twice and resuspended in lysis buffer (50 mM Tris-HCl, pH 7.4, 1 mM EDTA, 1 mM EGTA, 5 mM MgCl_2_, 150 mM NaCl, 1% Triton, 1 mM PMSF, 1 mM VO_4_, 100× Aprotinin, and 50× LAP, all purchased from Sigma, Milan, Italy, except LAP, which was obtained from Roche, Milan, Italy). The cell lysates were analyzed using SDS-PAGE with a standard running buffer (25 mM Trizma, 192 mM Glycine, and 0.1% SDS) and were transferred onto nitrocellulose membranes (Whatman Gmbh, Dassel, Germany) using transfer buffer (25 mM Trizma, 192 mM Glycine, 0.1% SDS, and 20% methanol, all purchased from Sigma-Aldrich, Milan, Italy). The membranes were blocked with 5% non-fat dry milk and were probed with goat anti-MxA, rabbit anti-ERK1/2 and mouse anti-pY-ERK1/2, (Santa Cruz, Milan, Italy), mouse anti-IFN-α (RD System, Milan, Italy), mouse anti-GAPDH, mouse anti-tubulin (Sigma-Aldrich, Milan, Italy), rabbit anti-TLR7, rabbit anti-MyD88, rabbit anti-pY- NF-κB, rabbit anti-NF-κB, mouse anti-pY-p38, rabbit anti-p38, rabbit anti-pY-JNK and rabbit anti- JNK (Cell Signaling, Euroclene, Milan, Italy) and mouse anti-HRS (Alexix Biochemicals, San Diego, CA, USA). The bands were visualized using ECL (GE Healthcare, Amersham, Buckinghamshire, UK) with exposure times of 2–10 min. The band intensity was evaluated by integrating all the pixels of the band after subtraction of the background to calculate the average of the pixels surrounding the band^9^.

### Immunoprecipitation

Lysates were prepared as described previously^9^, and the protein concentration was measured using a Bio-Rad protein assay kit (Munchen, Germany). Equal amounts of the cell lysates (2 mg of protein) were used for immunoprecipitation. MyD88 was immunoprecipitated for 24 h using the rabbit anti-MyD88 polyclonal antibody (Cell Signaling Euroclone, Milan Italy). EGFR was immunoprecipitated for 24 h using the anti-EGFR polyclonal antibody (Cell Signaling Euroclone, Milan, Italy). Following immunoprecipitation, the complexes were isolated using protein A/G magnetic beads and were washed 3× in lysis buffer. The pellet was resuspended in 35 μl of 2× Laemmli buffer (Sigma, Milan, Italy). The samples were heated to 95–100 °C for 5 minutes and were micro centrifuged for 1 minute at 14,000 × g. The samples were loaded (30 μl) onto SDS-PAGE gels (10%). The proteins were transferred to nitrocellulose membranes (Whatman Gmbh, Dassel, Germany), and the blots were probed for TLR7 with anti-TLR7 (Santa Cruz Biotech, Milan, Italy), for MyD88 with mouse anti-MyD88 (Abcam, Milan, Italy) and for EGFR with anti-EGFR polyclonal antibody (Cell Signaling Euroclone, Milan, Italy).

### PCR analysis

cDNAs were generated from the total RNA using the High Capacity cDNA Reverse Transcription Kit (Applied Biosystems, Foster City, CA, USA)^9^. The resulting cDNA samples were subjected to cycles of PCR amplification, followed by real-time PCR using TaqMan® preAmp Master Mix Kit protocol (Applied Biosystems, PN 4366127, Foster City, CA, USA). Each TaqMan Gene Expression assay consisted of two sequence-specific PCR primers and a TaqMan assay-FAM dye-labelled MGB probe. Eighty nanograms of total cDNA was used for each replicate assay. Three replicates were run for each sample in a 96-well plate format. The endogenous control gene used was beta-2-microglobulin (B2 M). The assays were run with 2× Universal PCR Master Mix without UNG (uracil-N-glycosylase) using an Applied Biosystems 7300 Real-Time PCR System and universal cycling conditions (10 min at 95 °C; 15 sec at 95 °C, 1 min at 60 °C for 40 cycles).

### Organ culture studies

For organ culture studies, biopsy fragments from the duodenum were obtained from CD patients with villous atrophy on GCD, controls, affected by gastroesophageal reflux, and CD patients on GFD. The age range for all subjects was 2 to 17 years. GFD patients had negative serology (anti-tTg antibodies between 0 and 1.5 U/ml and EMA negative) and normal biopsy (Marsh T0–1). GCD-CD patients with villous atrophy (Marsh T3a-c) had a positive serology (anti-tTg antibodies >30 U/ml and EMA positive) (Table 1). Anti-tTg antibodies were measured using the Eurospital kit, EU-tTG. Biopsy fragments were used to evaluate the expression of the MxA and IFN-α proteins using Western blotting. Intestinal biopsies from the GCD CD patients, the GFD CD subjects and control subjects were cultured^9^ with P31–43 (100 μg/ml) or P57–68 (100 μg/ml) or medium alone for 24 h to examine MxA and IFN-α expression.

**Table 1:** Organ culture studies subjects.

	Age (years)	SEX	BIOPSY (Marsh Classification**)	Serum Anti-TG2 (U/ml)	EMA
Controls (N = 21)	2–17	M = 7 F = 14	19: T0 2: T1	18: ND* 3: 0–1.5	19: ND* 2: negative
GCD-CD (N = 26)	2–15	M = 12 F = 14	2: T3a 16: T3c 8: T3c/b	26: 30–700	26: positive
GFD-CD (N = 6)	4–17	M = 3 F = 3	6: T0	6: 0–3.4	6: negative

*Not Determined. **T0: Normal; T1: Infiltrative lesion; T3: Flat destructive lesion (a: mild, b: moderate; c: total).

### Ethical statements

The protocol for this study was approved by the Ethical Committee of the University “Federico II”, Naples, Italy (ethical approval: C.E. n. 230/05). Biopsy fragments for organ culture studies were obtained from the duodenum of untreated patients with active and remission CD and controls affected by gastroesophageal reflux. Written informed consent was obtained from patients or from the next of kin, caretakers, or guardians on behalf of the minors/children participants involved in our study. The Authors confirm that all methods were performed in accordance with the relevant guidelines and regulations.

### Statistical analyses

Graphpad prism 5 (Graphpad Software, San Diego, CA, USA) was used for statistical analysis and graphic representation. Statistical analyses of differences were performed using Student’s *t*-test. A *p* value < 0.05 was considered statistically significant. One-way Anova, Bonferroni corrected (*p* value < 0.05 was considered statistically significant) was used for multiple comparisons as indicated in the figure legends.

## Electronic supplementary material

Supplemental Information
