# Supplementary material for: P31–43, an undigested gliadin peptide, mimics and enhances the innate immune response to viruses and interferes with endocytic trafficking: a role in celiac disease
Source: Sci Rep. 2018 Jul 17;8:10821. doi: 10.1038/s41598-018-28830-y (PMC6050301; doi:10.1038/s41598-018-28830-y)

Online Supplemental Material

P31-43, an undigested gliadin peptide, mimics and enhances the innate immune response to viruses and interferes with endocytic trafficking: a role in celiac disease.

Merlin Nanayakkara, Giuliana Lania, Mariantonia Maglio, Renata Auricchio, Cristiana De Musis, Valentina Discepolo, Erasmo Miele, Bana Jabri*, Riccardo Troncone, Salvatore Auricchio, and Maria Vittoria Barone°.

Supplemental Figure 1.

ERK activation by P31-43, permuted peptides and P57-68 in CaCo2 cells. A. The specificity of the effects of P31-43 was determined by western blotting (WB) lysates from CaCo2 cells treated with the indicated compounds for 30 min or with the mutated and not mutated P31-43 at a concentration of 100 μg/ml. Cell lysates were prepared as described in the main text, and the phosphorylated form of Erk [pY-Erk] was detected using an anti-pY-Erk E-4 mouse monoclonal antibody (Clone E4, Santa Cruz Biotechnology, Milan, Italy). Total Erk was detected using the rabbit polyclonal anti-Erk K23 antibody (clone K23, Santa Cruz Biotechnology, Milan, Italy). Statistical analyses were performed on the results from 3 independent experiments, and representative WB are shown. UN = untreated. Student's t-test compared to the UN sample. * p< 0.05. Sequences of the permuted peptides are shown on the right. B. P31-43 and P57-68 were not toxic following the application of 100 μg/ml for up to 6 hours. The toxicity of P31-43 was analysed using trypan blue staining, a vital colorant. Cells were automatically counted using the Countess™ Automated Cell Counter (Thermo Fisher Scientific, Milan, Italy). Statistical analyses were performed on results from 3 independent experiments using Student's t-test. C. Dose-dependent effects of P31-43 and P57-68 on ERK phosphorylation. P57-68 did not effectively inhibit ERK phosphorylation at 50 or 100 μg/ml. WB of CaCo2 cells lysates treated with the indicated compounds for 30 min. Cell lysates and blots were prepared as described in the main text; the statistical analysis was performed on results from 3 independent experiments, and representative WB are shown. Student's t-test compared to the NT sample. * p< 0.05.

Supplemental Figure 1

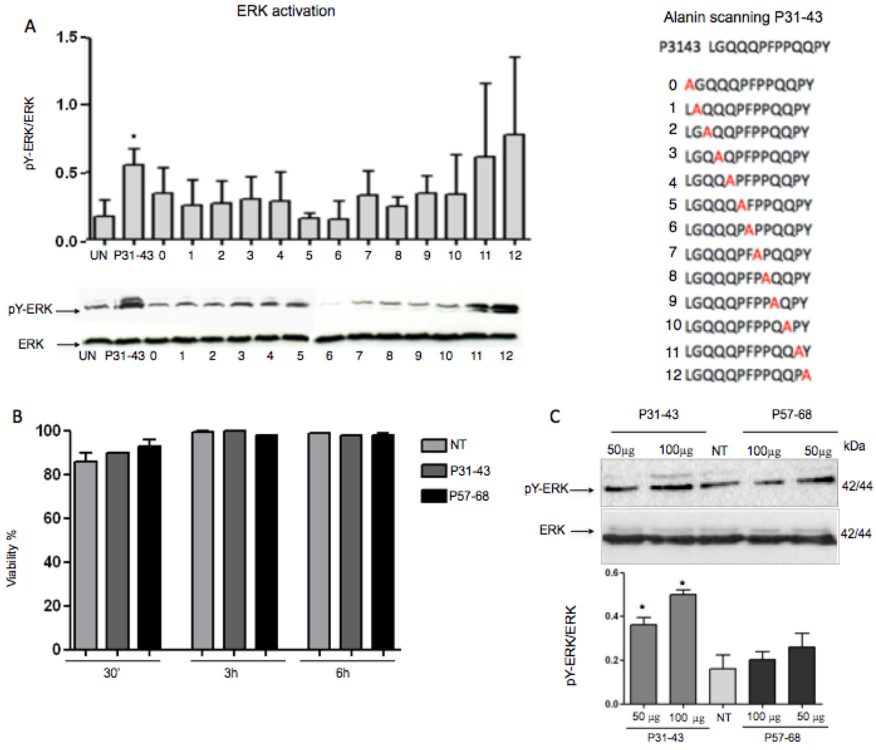

Supplemental Figure 2. The control A-gliadin peptide P57-68 did not activate the TLR7 pathway in CaCo2 cells. A. P57-68 did not increase the formation of the MyD88/TLR7 complex. (a) Western blot analysis of immuno-precipitated MyD88 in CaCo-2 cells. The upper panel was blotted with anti-TLR7 antibodies and the second panel was blotted with anti-MyD88 antibodies. The indicated treatments were applied for 30'. The results are representative of 3 independent experiments. b) Densitometric analysis. The relative amounts of TLR7 in the complex were normalized to the levels of immuno-precipitated MyD88. Columns represent the means and bars represent the standard deviations of 3 independent experiments. NT = not treated. B. Levels of the TLR7 and MyD88 proteins did not increase after treatment with P57-68. (a) Western blot analysis of protein lysates from CaCo-2 cells treated with the indicated compounds. The upper panels were blotted with anti-TLR7 antibodies, the second panel was blotted with anti-MyD88 antibodies and the third panel was blotted with anti-tubulin antibodies as a loading control. The results are representative of 3 independent experiments. (b) Densitometric analyses. The relative TLR7 and MyD88 levels were normalized to the tubulin levels. The treatments are indicated. Columns represent the means and bars represent the standard deviations of 3 independent experiments. NT = not treated. Student's t-test. C. P57-68 did not increase the MxA levels in CaCo-2 cells. (a) Western blot analysis of protein lysates from CaCo-2 cells treated with P57-68. The upper panel was blotted with anti-MxA antibodies and the second panel was blotted with anti-tubulin antibodies as a loading control. The results are representative of 3 independent experiments. (b) Densitometric analysis. The relative MxA levels observed after P57-58 treatment for 30' were normalized to tubulin. Columns represent the means and bars represent the standard deviations of 3 independent experiments. D. P57-68 did not increase the levels of the IFN-α 7 (a) and 17 (b) mRNAs. Quantitative PCR analysis of the IFN-α 7 and 17 mRNAs in CaCo-2 cells treated ON with P57-68. RQ = relative quantities of IFN-α 7 and 17 mRNAs. Columns represent the means and bars represent the standard deviations of 3 independent experiments. NT= not treated. E. P57-68 did not activate NF-κB. (a) Western blot analysis of protein lysates from CaCo-2 cells treated with the

indicated compounds for 30'. The upper panel shows levels of the phosphorylated form of NF-ϰB (pY-NF-ϰB), and the second panel shows levels of the total NF-ϰB protein. The results are representative of 3 independent experiments. (b) Densitometric analysis. The relative pY-NF-ϰB levels were normalized to the total NF-ϰB levels. The treatments are indicated. Columns represent the means and bars represent the standard deviations of 3 independent experiments. NT = not treated.

Online Supplemental Material

Supplemental Figure 2

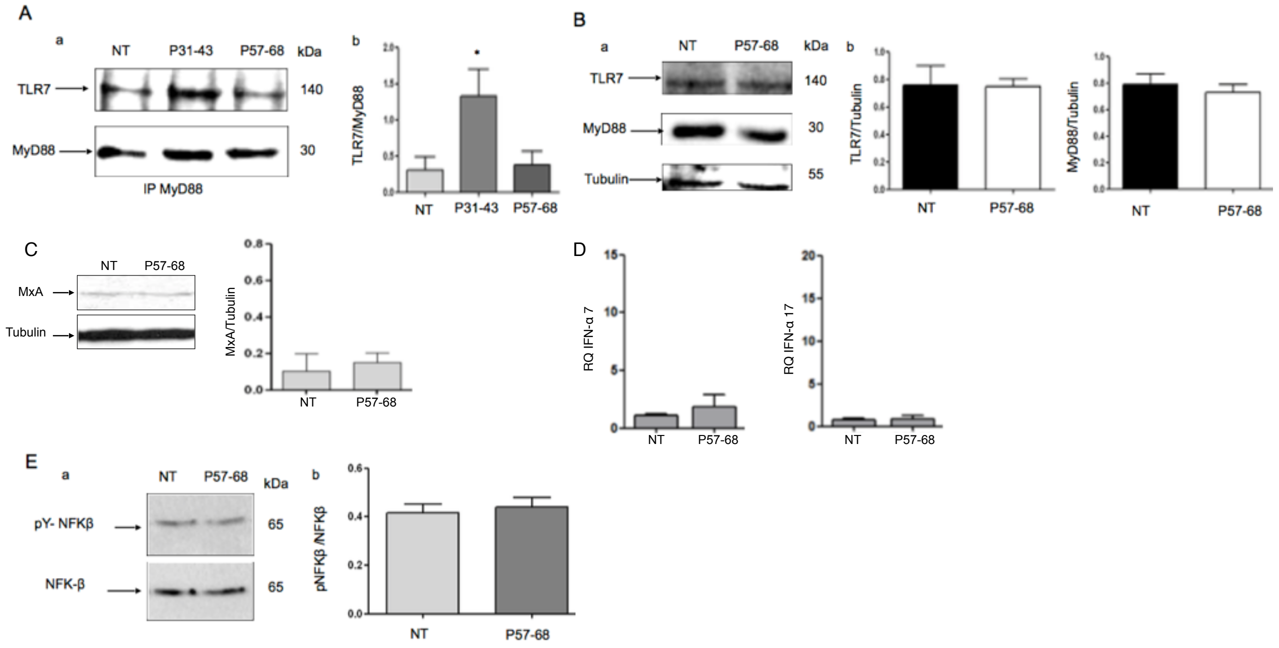

Supplemental Figure 3.

Similar to the viral ligand LOX, P31-43 induced the formation of the EGFR/TLR7 complex. A. Western blot analysis of immuno-precipitated EGFR in CaCo-2 cells. The upper panel was blotted with anti-TLR7 antibodies and the second panel was blotted with EGFR antibodies. Cells were treated with P31-43 (100 µg/ml) and LOX (1 mM) for 3 h. The results are representative of 3 independent experiments. B. Densitometric analysis. The relative amounts of TLR7 in the complex were normalized to the levels of immuno-precipitated EGFR. Columns represent the means and bars represent the standard deviations of 3 independent experiments. NT= not treated. Student's t-test compared with the NT sample. ** p < .01.

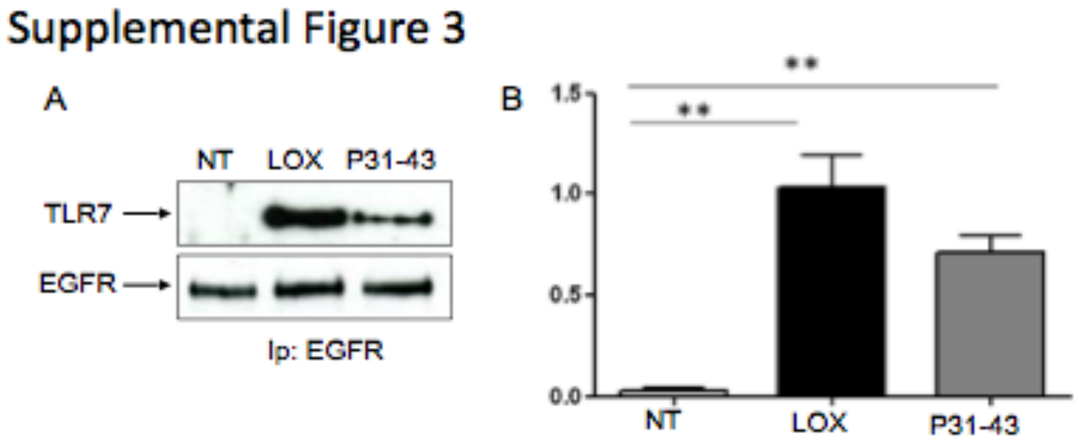

Supplemental Figure 4.

P31-43 and LOX treatments, as well as si-HRS, delay TLR7 trafficking in the early endocytic compartment. Co-localization of TLR7 with the early (EEA1) and late (LAMP2) vesicular markers in CaCo-2 cells before and after treatment with P31-43 or LOX or transfection with si-HRS. Treatments with P31-43 and LOX for 3 h or transfection with si-HRS induced a delay in TLR7 trafficking from the early vesicular compartment. A. Images of immunofluorescence staining in CaCo-2 cells treated with P31-43, LOX and si-HRS. TLR7 staining is shown in red, EEA1 staining is shown in green, and the yellow colour of the merged panels indicated co-localization. Nuclei are labelled blue. A 63× objective was used. Representative images from 3 similar independent experiments. B. Statistical analysis of the co-localization coefficients of TLR7 with EEA1. The control siRNA was ineffective (not shown). Columns represent the means and bars represent the standard deviations of 3 independent experiments. NT = not treated. Student's *t* test compared to the NT sample. * $p<0.05$. C. Images of immunofluorescence staining in CaCo-2 cells treated with P31-43, LOX and si-HRS. TLR7 staining is shown in red, LAMP2 staining is shown in green, and the yellow colour of the merged panels indicates co-localization. Nuclei are labelled blue. A 63× objective was used. Representative images from 3 similar independent experiments. D. Statistical analysis of the co-localization coefficients of TLR7 with LAMP2. Columns represent the means and bars represent the standard deviations of 3 independent experiments. NT = not treated. Student's t-test compared with the NT sample. * $p<0.05$.

Supplemental Figure 4

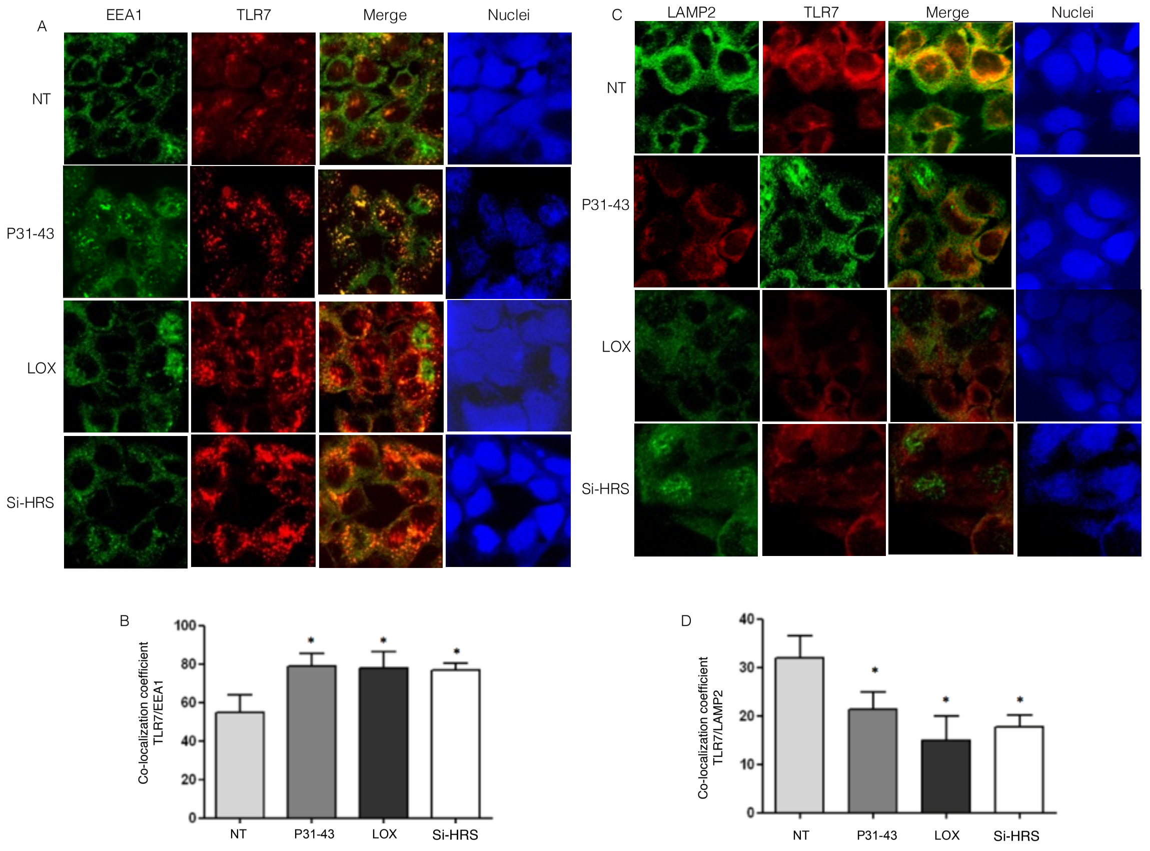

Supplemental Figure 5.

P31-43, but not P56-68, treatment delayed TLR7 trafficking in EEA1-positive early vesicles in CaCo2 cells. Time course experiments of TLR7 trafficking after P31-43 and P57-68 treatments. A. Statistical analysis of the co-localization coefficients of TLR7 with the early endocytic vesicle marker (EEA1). Treatments and times are indicated. Columns represent the means and bars represent the standard deviations of 3 independent experiments. NT = not treated. Student's t-test, * p<0.05. B. Statistical analysis of the co-localization coefficients of TLR7 with the late endocytic vesicle marker LAMP2. Treatments and times are indicated. Columns represent the means and bars represent the standard deviations of 3 independent experiments. NT = not treated. Student's t-test, * p<0.05.

Supplemental Figure 5

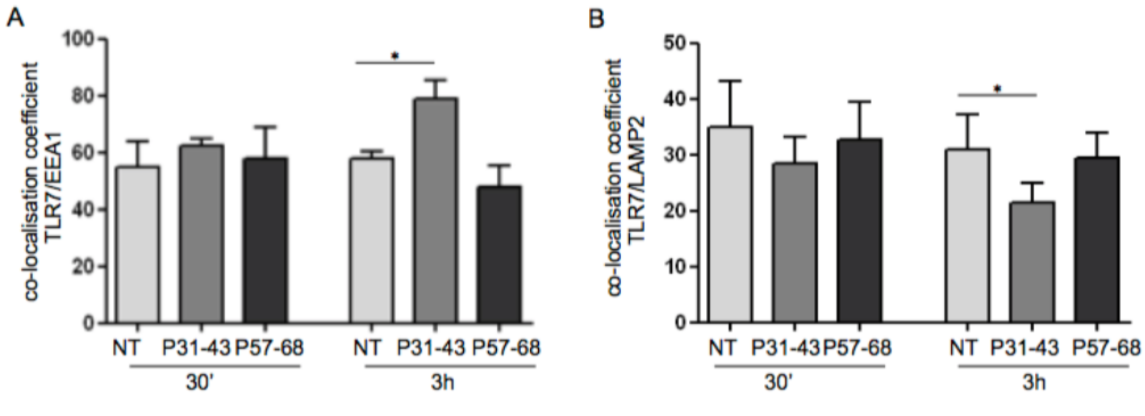

Supplement: Supplementary file 1 — Supplemental Information [file 41598_2018_28830_MOESM1_ESM.pdf]
